# Mitochondria as a Potential Regulator of Myogenesis

**DOI:** 10.1155/2013/593267

**Published:** 2013-02-03

**Authors:** Akira Wagatsuma, Kunihiro Sakuma

**Affiliations:** ^1^Graduate School of Information Science and Technology, The University of Tokyo, 7-3-1 Hongo, Bunkyo-ku, Tokyo 113-8656, Japan; ^2^Research Center for Physical Fitness, Sports and Health, Toyohashi University of Technology, 1-1 Hibarigaoka, Tenpaku-cho, Toyohashi 441-8580, Japan

## Abstract

Recent studies have shown that mitochondria play a role in the regulation of myogenesis. Indeed, the abundance, morphology, and functional properties of mitochondria become altered when the myoblasts differentiate into myotubes. For example, mitochondrial mass/volume, mtDNA copy number, and mitochondrial respiration are markedly increased after the onset of myogenic differentiation. Besides, mitochondrial enzyme activity is also increased, suggesting that the metabolic shift from glycolysis to oxidative phosphorylation as the major energy source occurs during myogenic differentiation. Several lines of evidence suggest that impairment of mitochondrial function and activity blocks myogenic differentiation. However, yet little is known about the molecular mechanisms underlying the regulation of myogenesis by mitochondria. Understanding how mitochondria are involved in myogenesis will provide a valuable insight into the underlying mechanisms that regulate the maintenance of cellular homeostasis. Here, we will summarize the current knowledge regarding the role of mitochondria as a potential regulator of myogenesis.

## 1. Introduction

Mitochondria generate most of the energy necessary for cellular function via oxidative phosphorylation (OXPHOS) as well as contribute to metabolism, Ca^2+^ signaling, and apoptosis. Besides, several lines of evidence suggest that mitochondrial function and activity are linked to cell differentiation, as have been shown in a wide variety of cell types including myoblasts [1–9]. When the myoblasts differentiate into myotubes, mitochondrial enzyme activity is drastically increased [10–12]. Likewise, muscle regeneration is also accompanied by an increased mitochondrial enzyme activity [13–15]. These findings suggest that the metabolic shift from glycolysis to OXPHOS as the major energy source occurs during myogenesis. The metabolic shift has been reported in embryonic stem cells (ESCs) [16, 17] and induced pluripotent stem cells (iPSCs) [18]. For example, iPSCs have low mitochondrial activity, relying predominantly on glycolysis for ATP generation and maintaining a state of dedifferentiation, while differentiation is accompanied by an increased mitochondrial activity [18]. Therefore, the metabolic shift may be a key event initiating cell differentiation. This shift requires an activation of mitochondrial biogenesis through coordinated expression of nuclear and mitochondrial genomes. Mitochondrial biogenesis is tightly controlled by transcriptional coactivators, transcription factors, and nuclear receptors [19–23]. Their expression is coordinately induced during myogenic differentiation [11, 12] and muscle regeneration [13, 15]. To further elucidate the relationship between mitochondria and cell differentiation, the effects of impairment of mitochondrial function and activity on myogenic cells have been investigated using antimycin [24], azide [24–27], chloramphenicol [4, 6–9], carbonyl cyanide m-chlorophenylhydrazone carbonyl (CCCP) [24], cyanide p-(trifluoromethoxy) phenylhydrazone (FCCP) [6], ethidium bromide (EtBr) [1–3, 24], myxothiazol [7], rhodamine 6G [28], rifampicin [1], rotenone [7], oligomycin [6, 7, 26], tetracycline [5], and valinomycin [24]. Overall, these antibiotics and chemicals can exert a negative influence on myogenesis. For example, respiration-deficient myoblasts devoid of mitochondrial DNA (rho° cells) by EtBr, an inhibitor of mtDNA replication and transcription, fail to differentiate into myotubes [1–3]. Rifampicin, which inhibits mitochondrial RNA synthesis, shows reversible inhibition of myotube formation [1]. Tetracycline, an inhibitor of mitochondrial protein synthesis, blocks myoblasts fusion [5]. Chloramphenicol, an inhibitor of mitochondrial protein synthesis, restricts myogenic differentiation [4, 6–9] and interferes with muscle regeneration [15]. Despite the data being accumulating, little is known about the molecular mechanisms underlying the regulation of myogenesis by mitochondria. In this paper, we will summarize the current knowledge regarding the role of mitochondria as a potential regulator of myogenesis.

## 2. Mitochondrial Biogenesis

Mitochondrial biogenesis (also referred to as mitochondriogenesis) is characterized as a vital process in the synthesis and degradation of the organelle [29, 30]. Therefore, this fundamental process comprehends (1) the synthesis import and incorporation of lipids and proteins to the existing mitochondrial reticulum; (2) the stoichiometric assembly of multisubunit protein complexes into a functional respiratory chain; (3) replication of the mitochondrial DNA (mtDNA); (4) selective degradation of mitochondria by autophagy (mitophagy) [22, 31, 32]. When it is not indicated, in this paper, mitochondrial biogenesis simply considers an increase in mitochondrial volume and changes in organelle composition per tissue or cell [31]. Mitochondrial biogenesis requires a coordination of expression of nuclear and mitochondrial genomes [20].

## 3. Transcriptional Regulation of Mitochondrial Biogenesis

Recent technological and scientific advances have allowed it to systematically identify the complement of over 1,000 different proteins that comprise the mammalian mitochondrial proteome [33]. The majority of the mitochondrial proteins are encoded by the nuclear genome and synthesized on cytoplasmic ribosomes [20, 34], whereas the minority are encoded and synthesized within the mitochondria. The mitochondrial genome contains 37 genes encoding 13 enzymes involved in OXPHOS, 22 types of transfer RNAs, and 2 types of ribosomal RNAs [35]. To maintain mitochondrial functionality, it is necessary for two genomes to be coordinately regulated [20]. It has been widely accepted that peroxisome proliferator-activated receptor gamma coactivator-1 alpha (PGC-1*α*) plays a central role in a regulatory network governing the transcriptional control of mitochondrial biogenesis [20–23]. PGC-1*α* works in concert with a wide variety of interacting partners, which are transcription factors and nuclear receptors [21, 22]. PGC-1*α* was discovered in a yeast two-hybrid screen for brown adipose-specific factors that interact with the nuclear receptor PPAR*γ* and are dramatically induced by exposure to cold in brown fat and skeletal muscle [36]. Subsequently, two additional PGC-1 family members were identified, PGC-1-related coactivator (PRC) [37] and PGC-1*β* [38]. The three coactivators regulate expression of a broad set of mitochondrial genes and promote mitochondrial biogenesis [39]. Since these coactivators lack DNA-binding activity [40, 41], PGC-1 family coactivators exert their effects through interactions with transcription factors and nuclear receptors bound to specific DNA elements in the promoter region of genes. For example, nuclear respiratory factor-1 (NRF-1) and NRF-2 (GA-binding protein; GABP) were the first regulatory factors implicated in the global expression of multiple mitochondrial functions in vertebrates [23]. Both NRF-1 and NRF-2 are involved in the transcriptional control of nuclear and mitochondrial genes involved in OXPHOS, electron transport (complex I-V), mtDNA transcription/replication, heme biosynthesis, protein import/assembly, ion channels, shuttles, and translation [19]. For more complete details, excellent review articles are already available on this subject [19–23].

## 4. Mitochondrial Enzyme Activity and Function during Muscle Regeneration and Myogenesis *In Vitro*

Muscle regeneration, which partially recapitulates embryonic myogenesis [42], would stimulate mitochondrial biogenesis [13]. Muscle injury was induced by intramuscular injection of either bupivacaine (which induces Ca^2+^ release from the sarcoplasmic reticulum (SR) and simultaneously inhibits Ca^2+^ reuptake into the SR, resulting in persistently increased [Ca^2+^] levels and leads to myofiber death), notexin (which involves Ca^2+^ overload and activation of Ca^2+^-dependent proteases, resulting in tissue necrosis), or freezing (which causes uniform and complete necrosis of myofibers). Such acute muscle injury shows a rapid loss of the activities of citrate synthase [13–15], a mitochondrial matrix enzyme participating in the Krebs cycle, which is often used as marker for the mitochondria content of a tissue, after 2-3 days, a time when degenerative myofibers still persist and proliferating myoblasts reside [13, 15]. The activity of citrate synthase then is increased drastically between days 5 and 10, a time when myoblasts differentiate into myotubes [13, 15]. Similarly, the rate of state-3 respiration (respiratory rate during active phosphorylation of ADP) is recovered [13]. The rate of state-4 respiration (respiratory rate after exhaustion of ADP) is comparable between control and injured muscles [13]. Accordingly, the pattern of changes in the respiratory control ratio (RCR), a measure of the “tightness of coupling” between electron transport and oxidative phosphorylation, is calculated as the ratio of state-3/state-4 respiration [43], closely resembling the pattern obtained with citrate synthase activity [13]. Consistent with the *in vivo* findings, the activity of mitochondrial enzymes including citrate synthase, isocitrate dehydrogenase, 3-hydroxyacyl CoA dehydrogenase, cytochrome *c* reductase, succinate dehydrogenase, and cytochrome oxidase is drastically increased during myogenic differentiation [10, 12]. The content of respiratory chain complexes is higher in myotubes than in myoblasts [11, 44]. Similarly, the rate of state-3 respiration exhibits the same trend observed in respiratory chain complexes [44]. Leary et al. examined the changes in metabolic rate during myogenic differentiation [25]. In proliferating myoblasts, approximately 30% of the ATP used by the cells is provided by OXPHOS, whereas terminally differentiated myotubes rely on mitochondrial respiration as their major source of metabolic energy (approximately 60%) [25]. Intriguingly, the total metabolic rate remains constant throughout the culture period, but there is a steady shift toward a greater reliance on mitochondrial pathways [25]. Taken together, these findings suggest that the metabolic shift from glycolysis to oxidative phosphorylation as the major energy source occurs during myogenesis.

## 5. Gene Expression Involved in Mitochondrial Biogenesis during Muscle Regeneration and Myogenesis *In Vitro*

An activation of mitochondrial biogenesis occurs through the coordinated expression of PGC-1 transcriptional coactivators, transcription factors, and nuclear receptors. Surprisingly, PGC-1*α* expression remains unchanged during muscle regeneration, whereas PRC and PGC-1*β* are upregulated 3 days after injury [15]. This finding may be in line with other studies using mouse myoblasts [37, 45, 46]. PGC-1*α* fails to detect in either myoblasts or myotubes, whereas PRC and PGC-1*β* are readily detectable [46]. In contrast, Duguez et al. have reported that PGC-1*α* is upregulated 10 days after injury [13]. Irrespective of whether PGC-1*α* is upregulated in injured muscle, these findings lead us to hypothesize that PRC and PGC-1*β* may contribute to the mitochondrial biogenesis, at least in part, at the early stage of muscle regeneration. It has been shown that PRC coactivates NRF-1 [37, 45], and PGC-1*β* also interacts with NRF-1 and estrogen-related receptor *α* (ERR*α*) [47], suggesting that both PRC and PGC-1*β* may functionally replace PGC-1*α* during muscle regeneration. In support of this hypothesis, several studies have revealed the potential roles of PRC and PGC-1*β* in mitochondrial biogenesis in myogenic cells. PRC-overexpressing myotubes show an elevated fatty acid oxidation and increased expression of mitochondrial genes [48]. Forced expression of PGC-1*β* in C2C12 cells results in increased mitochondrial biogenesis and oxygen consumption [49]. Skeletal muscle-specific PGC-1*β* transgenic mice exhibit increased mtDNA amount, mitochondrial content, mitochondrial enzyme activity, upregulation of mitochondrial genes, and enhanced exercise performance [50]. On the other hand, mice lacking PGC-1*β* show a reduced number of mitochondria, decreased respiration function, and decreased expression of mitochondrial genes [51]. However, the possibility cannot be excluded that PGC-1*α* may contribute to the mitochondrial biogenesis during muscle regeneration, as has been shown in gain-of-function and loss-of-function studies [52–56]. Accordingly, further studies are required to elucidate the role of PGC-1*α* in mitochondrial biogenesis during muscle regeneration. 

Not only PGC-1 family coactiuators but also NRF-1, NRF-2, and mitochondrial transcription factor A (TFAM) are also upregulated during muscle regeneration [15]. This is in line with the findings that PGC-1 stimulates an induction of NRF-1 and NRF-2 gene expression and can also interact directly with and coactivate NRF-1 on the promoter for TFAM [57]. TFAM plays a key role in mammalian mtDNA transcription/replication [21]. Likewise, when myoblasts differentiate into myotubes, PGC-1*α*, NRF-1, and TFAM are upregulated, and mtDNA content and copy number are increased 2–4-fold in myotubes relative to myoblasts [11, 12]. Therefore, upregulation of these genes contributes to increase the template availability for transcription and translation of key mitochondrial proteins necessary for myogenesis. 

## 6. Possible Role of Mitochondria in Regulating Muscle Regeneration

Recent studies have extended our knowledge of the potential role of mitochondrial biogenesis in muscle regeneration [15, 58]. It has been reported that muscle regeneration is impaired when mitochondrial protein synthesis is inhibited with chloramphenicol [15]. Chloramphenicol inhibits protein synthesis in mitochondria but not in mammalian cytoplasmic ribosomal systems [59] since mammalian mitochondrial ribosomes are susceptible to peptidyl-transferase inhibition by it [60]. Chloramphenicol reversibly binds to the 50S subunit of the 70S ribosome and blocks prokaryotic protein translation primarily by inhibiting peptidyl-transferase and blocking elongation [61]. Consequently, chloramphenicol inhibits the proper assembly of 4 out of 5 respiratory chain complexes within mitochondria and therefore potentially attenuates mitochondrial biogenesis in mammalian cells. Mice were intramuscularly injected with chloramphenicol at days 3, 5, and 7 after the initial freeze injury, and the muscle specimens were histochemically analyzed at day 10. Impairment of mitochondrial activity induced by chloramphenicol results in poor muscle regeneration with small myofibers and increased connective tissues [15]. Overall, this supports *in vitro* data that show that chloramphenicol blocks myogenic differentiation [4–9]. Therefore, *in vivo* data, when combined with the previous data *in vitro*, suggests a role for mitochondrial biogenesis for sustaining muscle regeneration. However, the molecular mechanisms remain unknown although chloramphenicol downregulates myogenin, which is required for terminal differentiation and myotube formation, in an avian QM7 myoblast [6, 8] and mouse C2C12 myoblast [9].

It has been reported that muscle regeneration is effectively accelerated using a method for complex mediated delivery to intracellular mitochondria [58]. The method is based on the mitochondriotropism of a multisubunit RNA import complex (RIC) [62]. Muscle injury was induced by piercing repeatedly with a 26-gauge hypodermic needle at an angle of ~45° to the longitudinal axis of the fiber, resulting in ~3000 myofibers being damaged at each insertion [58]. When a combination of polycistronic RNAs encoding the guanine-rich heavy-strand (H-strand) of the mitochondrial genome is administrated to injured muscle, it rejuvenates mitochondrial mRNA levels, organellar translation, respiratory capacity, and intramuscular ATP levels with reduced intracellular reactive oxygen species levels [58]. It increases proliferative potential of satellite cells and differentiation capacity of myoblasts concomitantly with upregulation of myogenic regulatory factors including Myf5, MyoD, myogenin, and MRF4, promoting muscle regeneration with the recovery of muscle contractility [58]. One of the most intriguing aspects of RIC-mediated transfection strategy, MyoD, and Numb-positive cells are detected and attached to old myofibers at the injury site [58]. This may provide new insight into the possible mechanism regulating muscle regeneration through enhancing mitochondrial activity. Numb protein has been generally considered to be a negative regulator of Notch signaling [63], which inhibits myogenic differentiation [63]. Numb segregates asymmetrically in dividing adult mouse muscle satellite cells [64, 65]. Attenuation of Notch signaling by Numb overexpression leads to the commitment of progenitor cells to the myoblast cell fate with increased expression of Myf5 and desmin [64]. Therefore, RIC-induced Numb protein may play a certain role in regulating muscle regeneration by modulating Notch signaling. However, recent evidence suggests that although forced expression of Numb in myogenic progenitors does not abrogate canonical Notch signaling, it can stimulate the self-renewal of myogenic progenitors [66]. Therefore, a role of Numb in regulating muscle regeneration remains to be elucidated. Furthermore, it is unknown how mitochondrial activity modulates Notch signaling at the present time. 

## 7. Do Mitochondria Act as a Potential Regulator of Myogenesis?

Korohoda et al. [4] have reported that chloramphenicol inhibits the fusion of myoblasts isolated from chick embryo skeletal muscle. This is among the first study to show the effect of chloramphenicol on myogenesis. They show that tryptose phosphate broth and nucleosides can restore the cell capacity to proliferate but not to fuse and differentiate in the presence of chloramphenicol [4]. Subsequently, it has been demonstrated that mitochondrial activity is an important regulator of myogenic differentiation in quail myoblasts of the QM7 cell line and mouse myoblasts of the C2C12 cell line using chloramphenicol [6, 8, 9]. Chloramphenicol-treated myoblasts proliferate at a slower rate than control myoblasts without inducing any alteration of cell viability [6]. When chronically exposed to chloramphenicol throughout the culture period, it severely suppresses myogenic differentiation [6, 8, 9]. The possibility can be excluded that intracellular ATP depletion induced by chloramphenicol could be responsible for the inhibition of myoblast differentiation for the following the reasons: (1) glycolysis fully compensates for mitochondrial impairment just before and during terminal differentiation, as shown in a marked accumulation of L-lactate in the culture medium [6], and this has been already reported in C2C12 cells using tetracycline [5]; (2) differentiation of myoblast is repressed especially when exposing to chloramphenicol at the onset of terminal differentiation [6]. These findings indicate that mitochondrial activity regulates myogenic differentiation independently of their implication in ATP synthesis [6].

Chloramphenicol inhibits myogenic differentiation by downregulating myogenin but not MyoD and Myf5 [6, 8]. Intriguingly, this downregulation is commonly observed in FCCP, myxothiazol [7], rotenone [7], and oligomycin [6, 7], which affect mitochondria at different levels. These findings suggest that myogenin could be an important target of mitochondrial activity. Chloramphenicol has no effect on myogenin mRNA stability [6], suggesting that mitochondrial activity could regulate myogenin expression at the transcriptional level [6]. Unexpectedly, overexpression of neither myogenin nor MyoD fails to restore differentiation capacity in chloramphenicol-treated myoblasts [6]. This indicates that mitochondrial activity could regulate myogenic differentiation by decaying ability of myogenic regulatory factors via other negative regulators. Chloramphenicol has no effect on the expression of MEF2C (myocyte enhancer factor 2C) and Id (inhibitor of differentiation) [8]. Seyer et al. have identified c-Myc (cellular myelocytomatosis oncogene) gene, which could be a target gene regulated by mitochondrial activity [8]. c-Myc is a proto-oncogene encoding a transcription factor [67], which plays a role in regulating myogenesis [68–74]. Impairment of mitochondrial activity by chloramphenicol abrogates the downregulation of c-Myc normally occurring at the induction of differentiation in control cells [8]. Overexpression of c-Myc mimics the influence of mitochondrial activity inhibition on myogenic differentiation [8]. A triiodothyronine-dependent mitochondrial transcription factor (p43) overexpression, which stimulates mitochondrial activity, downregulates c-Myc expression [8]. These findings suggest the possibility that c-Myc could be a primary target of mitochondrial activity. Indeed, the endogenous c-Myc is downregulated within the first 24 h after switching to a differentiation medium [70]. Ectopic expression of c-Myc in quail myoblasts fails to form myotubes and downregulates MyoD, myogenin, and Myf5 expression [73]. Cotransfection of c-Myc with MyoD and myogenin in NIH 3T3 cells inhibits myogenic differentiation [71]. 

While these findings are compelling, a role of c-Myc should be carefully considered. First, irreversible repression of c-Myc is not required for terminal myogenic differentiation, and its expression is insufficient to suppress the differentiated phenotype, since nuclear runoff transcription assay demonstrates that c-Myc and skeletal muscle-specific genes could be simultaneously transcribed in both biochemically differentiated cells (no fusion) and terminally differentiated cells [69]. The c-Myc- transformed C2C12 cells retain the ability to undergo commitment and biochemical differentiation, but they are strikingly unable to fuse into multinucleated myotubes with no change in the expression of MyoD, myogenin, and myosin heavy chain [72]. These findings lead us to rethink how c-Myc modulates myogenic differentiation. Secondly, c-Myc represses p21^Cip1/WAF1^ expression through transcriptional activator, Miz-1- (c-Myc interacting zinc-finger protein 1-) dependent interaction with p21^Cip1/WAF1^ core promoter [75]. In addition, c-Myc interacts with Miz-1 and recruits DNA methyltransferase 3A to p21^Cip1/WAF1^ promoter to silence p21 transcription [76]. The expression of p21^Cip1/WAF1^ is known to be a key event triggering the withdrawal of myoblasts from the cell cycle to G_0_, a prerequisite to myogenic differentiation [77]. Indeed, chloramphenicol and overexpression of c-Myc decrease the proportion of myoblasts in the G_0_-G_1_ phase, whereas overexpression of p43 exerts opposite influence [8]. These findings suggest the possibility that mitochondrial activity could regulate myoblast cell cycle withdrawal by modulating expression of p21^Cip1/WAF1^ through c-Myc/Miz-1 complex. Thirdly, Myc is a member of the Myc/Max (Myc-associated factor X)/Mad (MAX dimerization protein) transcriptional network that comprises a group of widely expressed transcription factors [78]. c-Myc/Max heterodimers transactivate its downstream genes by binding to the E-box sequence 5′-CACGTG-3′ in the target promoter, whereas Mad/Max heterodimers act as transcriptional repressors at the same E-box-related DNA-binding sites [78]. Therefore, c-Myc/Max heterodimers function by competing with Mad/Max heterodimers, resulting in controlling the expression of their target genes. Intriguingly, a switching from c-Myc/Max to Mad/Max heterodimers occurs when leukemia cells differentiate into monocyte/macrophage [79, 80]. These findings lead us to hypothesize that mitochondrial activity may be involved in this switching during myogenic differentiation. It requires additional studies to validate this observation in myogenic cells. Finally, a new mode of Myc regulation has been recently reported in myogenic differentiation [81]. Myc protein is cleaved by a calpain to generate a cytoplasmic form, “Myc-Nick,” which retains Myc box regions but lacks nuclear localization sequence and the basic helix-loop-helix/leucine zipper domains essential for heterodimerization with Max and DNA binding activity [81]. During myogenic differentiation, while the full-length Myc decreases, Myc-nick is increased. Ectopic expression of Myc-nick in human primary myoblasts, human rhabdomyosarcoma (RD) cells, and mouse C2C12 myoblasts accelerates their differentiation and increases expression of skeletal muscle-specific markers [81]. Taken together, the mechanisms underlying the regulation of biological function of c-Myc are complicated. Therefore, further studies are needed to elucidate the role of c-Myc in the regulation of myogenesis by mitochondria.

 To further understand the molecular mechanisms underlying the regulation of myogenic differentiation by mitochondria, Seyer et al. [9] conducted a comprehensive differential display analysis using total RNA from control and chloramphenicol-treated myoblasts to search for other gene modulating by mitochondrial activity [9]. They identified calcineurin (also referred to as protein phosphatase 2B) as another candidate molecule [9], in which serine/threonine protein phosphatase under the control of a eukaryotic Ca^2+^- and calmodulin plays a critical role in the coupling of Ca^2+^ signals to cellular responses [82]. It is a heterodimeric enzyme consisting of a 60 kDa catalytic A subunit (calcineurin A) and 19 kDa calcium-binding regulatory B subunit (calcineurin B) [82]. Calcineurin signaling has been implicated in regulating myogenesis [83–90]. Chloramphenicol attenuates the differentiation-induced upregulation of calcineurin A, whereas overexpression of p43 increases calcineurin A expression in proliferating myoblasts [9]. Based on these findings, they suggest that calcineurin could be a novel target regulated by mitochondrial activity. Intriguingly, expression of a constitutively active form of calcineurin upregulates the expression of myogenin [85]. Calcineurin regulates expression of the myogenin gene at the transcriptional level by activating MEF2 and MyoD transcription factors [87]. Taken together, mitochondrial activity may regulate myogenesis through calcineurin-mediated myogenin expression. On the other hand, it has been shown that calcineurin A and its direct downstream transcriptional effector, NFATc (nuclear factor of activated T-cells), are upregulated concomitantly with a modest increase in calcineurin B in mtDNA-depleted cells (only ~20% of the mtDNA content compared with normal untreated cells) [24]. Biswas et al. developed myogenic cell lines with partially depleted mtDNA when chronically exposed to EtBr for many passages to investigate the mechanism of mitochondrial-nuclear crosstalk [24]. The mtDNA-depleted cells have an elevated steady-state cytosolic Ca^2+^ level ([Ca^2+^]i), as shown in other mitochondrial inhibitors including antimycin, azide, CCCP, and valinomycin [24]. Therefore, increased cytosolic Ca^2+^ may stimulate the expression of calcineurin-related molecules in the myoblasts treated with these drugs. It is to be noted that increased expression of calcineurin is observed by mtDNA depletion or acute treatment (30 min) with high amounts of mitochondrial inhibitors. As already described, mtDNA-depleted myoblasts by EtBr fail to differentiate into myotubes [1–3], and NFAT is not an essential downstream target of calcineurin during myogenesis [85]. Therefore, the activation of calcineurin pathway induced by impairment of mitochondrial function and activity could not contribute to myogenesis. 

The nuclear factor-*κ*B (NF-*κ*B) functions as a negative regulator of myogenesis [91]. NF-*κ*B is a heterodimeric or homodimeric complex formed from five distinct subunits: RelA (p65), RelB, c-Rel, NF-*κ*B1 (p50/p105), and NF-*κ*B2 (p52/p100) [91]. Only RelA, c-Rel, and RelB possess C-terminal transcriptional transactivation domains, whereas NF-kB1 and NF-kB2 lack intrinsic transactivating properties and instead function as homodimeric transcriptional repressors or modulators of transactivating dimer partners [91]. When stimulated by a wide variety of different stimuli, I*κ*B is phosphorylated by I*κ*B kinase (IKK) complex and subsequently degraded by the proteasome, allowing NF-*κ*B to translocate into the nucleus where they regulate target gene expression [91]. Respiration-deficient myoblasts devoid of mitochondrial DNA by EtBr show a decreased expression of RelA, increased expression of I*κ*B and p50, and unchanged expression of RelB and p52 [24]. Intriguingly, other mitochondrial inhibitors also have same effects on their expression [24]. These findings suggest that mitochondrial activity can modulate NF-*κ*B transcriptional activity although it is required for measuring its DNA binding activity, for example, by an electrophoretic mobility shift assay. 

## 8. Conclusion

**Figure 1:**
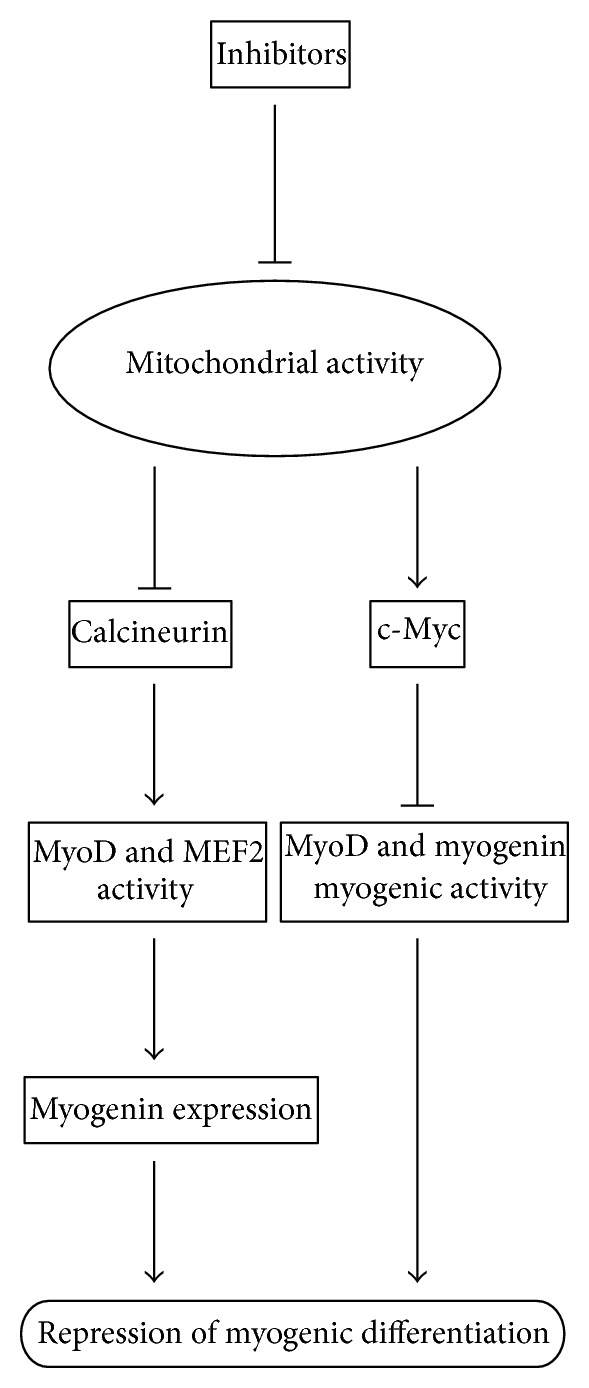
Hypothetic model of mitochondrial activity in myogenic differentiation.

This paper provides the current knowledge about the role for mitochondria as a potential regulator of myogenesis. Several studies have highlighted that mitochondria play a role in regulating myogenic differentiation possibly through a number of mechanisms. In particular, myogenin, c-Myc, and calcineurin have been identified as candidate molecules of mitochondrial target [6, 8, 9]. Together with previous data [8, 9, 87], a hypothetical model involving c-Myc and calcineurin in the regulation of myogenic differentiation by mitochondrial activity is presented in Figure 1. In this model, when myoblasts are induced to differentiate in the presence of mitochondrial inhibitors, downregulation of c-Myc could be inhibited, which depresses the activity of MyoD and myogenin, resulting in blocking myogenic differentiation. Decreased calcineurin signaling by inhibiting mitochondrial activity could contribute to myogenin expression through modulating MyoD and MEF2 activity. Understanding how mitochondria are involved in myogenesis will provide a valuable insight into the underlying mechanisms that regulate the maintenance of cellular homeostasis. Recently, it has been reported that the transgenic mice with skeletal muscle-specific expression of PGC-1*α* preserve mitochondrial function as well as neuromuscular junctions and muscle integrity during ageing [92], and mitochondrial gene therapy may be effective in the treatment of muscle injury [58]. These efforts may facilitate to understand the molecular mechanisms of mitochondrial disorders.
